# Risk factors for adenocarcinoma and squamous cell carcinoma of the cervix in women aged 20–44 years: the UK National Case–Control Study of Cervical Cancer

**DOI:** 10.1038/sj.bjc.6601296

**Published:** 2003-11-25

**Authors:** J Green, A Berrington de Gonzalez, S Sweetland, V Beral, C Chilvers, B Crossley, J Deacon, C Hermon, P Jha, D Mant, J Peto, M Pike, M P Vessey

**Affiliations:** 1 Cancer Research UK Epidemiology Unit, University of Oxford, Gibson Building, Radcliffe Infirmary, Oxford OX2 6HE, UK; 2 Research and Development Office, Department of Health, Nottingham, UK; 3 Institute of Cancer Research, Sutton, Surrey, UK; 4 Department of Public Health Sciences, University of Toronto, Ontario, Canada; 5 Department of Primary Health Care, Institute of Health Sciences, Oxford, UK; 6 London School of Hygiene and Tropical Medicine, London, UK; 7 Department of Preventive Medicine, Keck School of Medicine, University of Southern California, USA; 8 Unit of Health Care Epidemiology, Department of Public Health, Institute of Health Sciences, Oxford, UK

**Keywords:** cervix neoplasms, risk factors, adenocarcinoma, squamous cell carcinoma

## Abstract

We report results on risk factors for invasive squamous cell and adenocarcinomas of the cervix in women aged 20–44 years from the UK National Case–Control Study of Cervical Cancer, including 180 women with adenocarcinoma, 391 women with squamous cell carcinoma and 923 population controls. The risk of both squamous cell and adenocarcinoma was strongly related to the lifetime number of sexual partners, and, independently, to age at first intercourse. The risk of both types of cervical cancer increased with increasing duration of use of oral contraceptives, and this effect was most marked in current and recent users of oral contraceptives. The risk of squamous cell carcinoma was associated with high parity and the risk of both squamous cell and adenocarcinoma increased with early age at first birth. Long duration smoking (20 or more years) was associated with a two-fold increase in the risk of squamous cell carcinoma, but smoking was not associated with the risk of adenocarcinoma. Further studies are needed to confirm the suggestion from this and other studies of differences in risk related to smoking between squamous cell and adenocarcinomas of the cervix.

Among cancers of the uterine cervix, adenocarcinomas are much less common than squamous cell carcinomas. As for squamous cell carcinoma, human papillomavirus (HPV) infection appears to be a major risk factor for adenocarcinoma of the cervix (Kjaer and Brinton, 1993; Walboomers *et al*, 1999; Santos *et al*, 2001; Altekruse *et al*, 2003), but evidence on other factors that may affect the risk of adenocarcinoma is limited and not entirely consistent (Parazzini and La Vecchia, 1990; Kjaer and Brinton, 1993; Ursin *et al*, 1994,^1996^; Lacey *et al*, 1999,^2000^,^2001^; Madeleine *et al*, 2001; Altekruse *et al*, 2003). The question of whether there are substantial differences in the risk factors for adenocarcinomas and squamous cell carcinomas remains unresolved. Few epidemiological studies have been designed specifically to provide a direct comparison between adenocarcinoma and squamous cell carcinoma of the cervix, and most studies of cervical carcinoma have had too few cases of adenocarcinoma to allow full evaluation of the possible risk factors. We present here results from the UK National Case–Control Study of Cervical Cancer, a case–control study of risk factors for invasive cervical cancer in women under 45 years old at diagnosis, with 180 cases of adenocarcinoma (including adenosquamous carcinoma), 391 cases of squamous cell carcinoma and 923 population controls. Information was available for all study subjects on sexual and reproductive factors, cervical screening, body weight, smoking and the use of barrier and hormonal contraceptives. Additional information was collected on HPV serology for a subgroup of subjects, and for this subgroup results have previously been published on the relationship between HPV positivity and risk of cervical cancer (Jha *et al*, 1993), and on oral contraceptive use as a risk factor for cervical cancer in relation to HPV positivity (Berrington *et al*, 2002).

## METHODS

Recruitment took place between 1987 and 1989. Cases were white women with biopsy-confirmed invasive cervical cancer diagnosed at age 20–44 years in the UK between 1984 and 1988, selected from five UK Cancer Registries (South Thames, Oxfordshire, Lothian, North Western and Yorkshire), with additional cases from hospital treatment centres. Squamous cell and adenocarcinoma cases were frequency matched by age group; the mean (and median) age of squamous cell carcinoma cases was 33 years and of adenocarcinoma cases was 35 years. For each woman with cervical cancer, between one and three control women matched for age (within 12 months) were selected from the patient lists of the case's general practitioner. Women were excluded from the study if they had prior malignancy (other than squamous cell and basal call carcinoma of the skin and intraductal breast cancer), severe mental handicap or mental illness, insufficient knowledge of the English language, were a member of the Armed Services or were born outside Europe, Africa, Australasia or North America.

Cases and controls were interviewed at home by a trained nurse interviewer. A structured questionnaire was used to obtain information on occupation, past medical history, smoking and sexual and reproductive factors, including use of barrier and oral contraceptives and history of screening for cervical cancer. Information on obstetric history and prescriptions for oral contraceptives was obtained from the notes of the general practitioner and, if relevant, the family planning clinic. Results of cervical smear tests were confirmed through the cervical screening service. A total of 181 women with adenocarcinoma or adenosquamous carcinoma and three matched controls per case (538 in total), and 397 women with squamous cell carcinoma, each with one matched control, were recruited. For analysis, subjects who were virgins and those with unknown values for any of the adjustment variables were excluded; final analyses were based on 180 cases of adenocarcinoma and their 532 matched controls, and 391 matched pairs of squamous cell carcinoma cases and controls.

In order to increase the stability of risk estimates, and to provide a better comparison between the two histological types, the matching was broken and analyses were performed using a single, pooled, group of controls. Odds ratios (ORs) and 95% confidence intervals (CIs) for the variables of interest were calculated separately for adenocarcinoma and for squamous cell carcinoma compared with controls, and for squamous cell carcinoma cases compared with adenocarcinoma cases, using unconditional multiple logistic regression. Tests for linear trends were obtained by using the midpoint values of each category and treating this scored variable as continuous. Potential confounders were investigated and the following were adjusted for, as appropriate, in all models: age (5-year age groups; finer stratification by age did not materially alter the results), recruitment centre, age at first intercourse, duration of oral contraceptive use, level of education, number of negative screening results, smoking status and total number of sexual partners.

## RESULTS

### Sexual behaviour

For both squamous cell carcinoma and adenocarcinoma, the risk of cervical cancer increased with increasing lifetime number of sexual partners (Table 1

**Table 1:** Odds ratios (ORs) and 95% confidence intervals (CIs) for adenocarcinomas and squamous cell carcinomas of the cervix in relation to sexual behaviour and body weight

	**Adenocarcinoma**			**Squamous cell carcinoma**			**Squamous *vs* adenocarcinoma**		
	**Cases/controls**	**OR_a_**	**95% CI**	**Cases/controls**	**OR_b_**	**95% CI**	**Squamous/adeno cases**	**OR_c_**	**95% CI**
*Lifetime number of sexual partners*									
1	52/417	1.00		60/417	1.00		60/52	1.00	
2–4	84/330	1.96	1.31–2.93	186/330	3.31	2.34–4.70	186/84	1.65	1.00–2.73
5+	44/176	1.98	1.21–3.26	145/176	4.09	2.75–6.08	145/44	1.78	1.00–3.18
*Trend test*			*P*=*0.1*			*P<0.0001*			*P*=*0.3*
									
*Lifetime number of regular sexual partners (3+ months)*^a^									
0 or 1	63/499	1.00		91/499	1.00		91/63	1.00	
2	60/223	1.97	1.29–3.02	111/223	2.30	1.62–3.27	111/60	1.01	0.61–1.67
3+	57/201	2.18	1.36–3.47	189/201	3.40	2.38–4.86	189/57	1.63	0.97–2.71
*Trend*			*P*=*0.003*			*P*<*0.0001*			*P*=*0.03*
									
*Lifetime number of nonregular sexual partners*^b^									
0	101/603	1.00		170/603	1.00		170/101	1.00	
1–4	66/251	1.26	0.85–1.87	163/251	1.39	1.02–1.89	163/66	1.09	0.70–1.70
5+	13/69	0.75	0.37–1.52	58/69	1.29	0.80–2.07	58/13	1.28	0.60–2.75
*Trend*			*P*=*0.3*			*P*=*0.5*			*P*=*0.5*
									
*Age at first intercourse (years)*									
20+	32/283	1.00		42/283	1.00		42/32	1.00	
18–19	57/297	1.41	0.86–2.32	103/297	1.43	0.93–2.20	103/57	1.03	0.55–1.92
⩽17	91/343	2.01	1.23–3.30	246/343	2.70	1.78–4.11	246/91	1.38	0.75–2.55
*Trend*			*P*=*0.004*			*P*<*0.0001*			*P*=*0.2*
									
									
*Weight at reference age (kg)*^c^									
<55	52/289	1.00		141/289	1.00		141/52	1.00	
55–64	82/359	1.25	0.84–1.86	151/359	0.95	0.70–1.30	151/82	0.71	0.45–1.13
65+	45/273	1.00	0.63–1.57	99/273	0.85	0.61–1.20	99/45	0.90	0.54–1.52
*Trend*			*P*=*0.7*			*P*=*0.4*			*P*=*0.8*
									
*Body mass index at reference age*^c^									
Normal (20–24.9)	104/540	1.00		228/540	1.00		228/104	1.00	
Overweight/obese (25+)	40/219	0.98	0.64–1.49	78/219	0.87	0.62–1.23	78/40	0.89	0.55–1.46
Underweight (<20)	35/162	1.16	0.74–1.81	85/162	1.11	0.79–1.55	85/35	0.90	0.54–1.49

OR_a_, odds ratio for adenocarcinoma *vs* controls; OR_b_, odds ratio for squamous cell carcinoma *vs* controls; OR_c_, odds ratio for squamous cell carcinoma cases *vs* adenocarcinoma cases; all adjusted for age, region, smoking, duration of oral contraceptive use, number of negative screening results and education and adjusted as appropriate for total number of sexual partners and age at first intercourse.

a Adjusted for nonregular partners instead of total partners.

b Adjusted for regular partners instead of total partners.

c Reference age=age 1 year before diagnosis (cases) or pseudodiagnosis (controls).

). The effect was somewhat stronger for squamous cell carcinoma, with an odds ratio of 4.09 (95% CI (2.75–6.08) for women reporting five or more partners compared to those with one partner. There was a highly significant trend in risk (*P*=<0.0001). The equivalent odds ratio for adenocarcinoma was 1.98 (1.21–3.26), and there was no significant trend (*P*=0.1). For both types of cervical cancer, the association with sexual partners was seen only for regular (relationship lasting more than 3 months) and not for non-regular partners. Odd ratios for cervical cancer in women with three or more regular partners compared with women with one or no regular partners were 3.40 (2.38–4.86) for squamous cell carcinoma and 2.18 (1.36–3.47) for adenocarcinoma. Direct comparison showed a higher risk of marginal statistical significance for squamous cell compared with adenocarcinoma for five or more total partners (OR=1.78 (1.00–3.18)) and for three or more regular partners (OR=1.63 (0.97–2.71)). Owing to the small numbers of adenocarcinoma cases in the upper categories for number of partners, these results are given for three categories only; finer stratification by number of partners within the upper category for lifetime or regular partners did not substantially alter the results.

The risk of cervical cancer was higher for earlier age at first intercourse for both squamous cell (*P* for trend ⩽0.0001) and adenocarcinoma (*P*=0.004). Women with an age at first intercourse of 17 years or less had a 2–3-fold higher risk of cancer than those with first intercourse at 20 years or older. Direct comparison showed no significant difference between squamous cell and adenocarcinoma cases.

### Weight and body mass index

No relationship was seen between either self-reported weight or body mass index (1 year before diagnosis or pseudodiagnosis) and the risk of squamous cell or adenocarcinoma of the cervix (Table 1).

### Reproductive factors

Of 391 women with squamous cell carcinoma, 350 had been pregnant, of whom 331 reported one or more live births, six reported one or two stillbirths and 152 reported one or more interrupted pregnancies (termination of pregnancy or spontaneous miscarriage). For 180 women with adenocarcinoma, the equivalent numbers were 163 ever pregnant, 159 reporting one or more live births, two reporting stillbirth and 54 reporting interrupted pregnancy. Table 2

**Table 2:** Odds ratios (ORs) and 95% confidence intervals (CIs) for adenocarcinomas and squamous cell carcinomas of the cervix in relation to reproductive factors

	**Adenocarcinoma**			**Squamous cell carcinoma**			**Squamous *vs* adenocarcinoma**		
	**Cases/controls**	**OR_a_**	**95% CI**	**Cases/controls**	**OR_b_**	**95% CI**	**Squamous/adeno cases**	**OR_c_**	**95% CI**
*Ever given birth*									
Never	21/150	1.00		60/150	1.00		60/21	1.00	
Ever	159/773	1.24	0.72–2.14	331/773	1.26	0.85–1.86	331/159	1.09	0.58–2.04
									
*Total number of live births*									
0	21/150	1.00		60/150	1.00		60/21	1.00	
1	38/176	1.27	0.69–2.34	62/176	0.88	0.55–1.40	62/38	0.73	0.36–1.49
2	65/377	1.14	0.63–2.05	145/377	1.41	0.92–2.17	145/65	1.33	0.67–2.63
3+	56/220	1.44	0.76–2.73	124/220	1.86	1.16–2.99	124/56	1.47	0.70–3.11
*Trend*			*P*=*0.3*			*P*=*0.001*			*P*=*0.1*
									
*Time since last birth*									
Never	21/150	1.00		60/150	1.00		60/21	1.00	
⩽10 years	77/468	1.13	0.65–1.99	219/468	1.30	0.88–1.94	219/77	1.24	0.65–2.37
10+ years	82/305	1.63	0.86–3.07	112/305	1.07	0.66–1.73	112/82	0.66	0.32–1.40
*Heterogeneity between groups within parous women*			*P*=*0.2*			*P*=*0.3*			*P*=*0.03*
									
*Age at first birth*									
Never	21/150	1.00		60/150	1.00		60/21	1.00	
25+ years	36/272	0.92	0.49–1.70	70/272	0.94	0.60–1.47	70/36	1.07	0.52–2.18
20–24 years	63/322	1.40	0.77–2.55	108/322	1.23	0.79–1.91	108/63	1.04	0.51–2.11
15–19 years	60/179	1.71	0.89–3.29	153/179	2.01	1.26–3.21	153/60	1.16	0.57–2.38
*Trend within parous women*			*P*=*0.03*			*P*=*0.002*			*P*=*0.7*
									
									
*Number of interrupted pregnancies*									
0	126/647	1.00		239/647	1.00		239/126	1.00	
1+	54/276	0.91	0.63–1.31	152/276	1.16	0.88–1.54	152/54	1.36	0.89–2.08

OR_a_, odds ratio for adenocarcinoma *vs* controls; OR_b_, odds ratio for squamous cell carcinoma *vs* controls; OR_c_, odds ratio for squamous cell carcinoma *vs* adenocarcinoma cases; all adjusted for age, region, total number of sexual partners, age at first intercourse, smoking, duration of oral contraceptive use, number of negative screening results and education.

shows odds ratios for the risk of adenocarcinoma and squamous cell carcinoma in relation to several reproductive factors. No statistically significant associations were seen between the risk of squamous cell or adenocarcinoma and ever having given birth, time since last birth or number of interrupted pregnancies. For squamous cell carcinoma only there was a statistically significant increase in risk associated with increasing number of live births (OR=1.86 (1.16–2.99) for three or more live births compared with no live births; *P* trend=0.001). For adenocarcinoma, the odds ratio for three or more live births was 1.44 (0.76–2.73), with no significant trend (*P*=0.3). Both squamous cell and adenocarcinoma showed an increased risk with early age at first birth, with women with age at first birth of 15–19 years having a risk of cervical cancer about twice that of women whose first birth was at 25 years or older. On direct comparison, there were no statistically significant differences between squamous cell carcinoma and adenocarcinoma cases in relation to reproductive factors.

### Oral contraceptives

Use of oral contraceptives was associated with an increased risk of cervical cancer (Table 3

**Table 3:** Odds ratios (OR) and 95% confidence intervals (CI) for adenocarcinomas and squamous cell carcinomas of the cervix in relation to contraceptive use

	**Adenocarcinoma**			**Squamous cell carcinoma**			**Squamous *vs* adenocarcinoma**		
	**Cases/controls**	**OR_a_**	**95% CI**	**Cases/controls**	**OR_b_**	**95% CI**	**Squamous/adeno cases**	**OR_c_**	**95% CI**
*Oral contraceptive (OC) use*									
Never^a^	33/232	1.00		62/232	1.00		62/33	1.00	
Ever	147/691	1.56	1.01–2.42	329/691	1.37	0.97–1.94	329/147	1.35	0.64–2.86
Past	108/543	1.42	0.91–2.23	244/543	1.31	0.92–1.87	244/108	0.97	0.56–1.66
Current	39/148	2.36	1.34–4.16	85/148	1.64	1.06–2.55	85/39	0.76	0.39–1.45
									
*Duration of OC use*									
Never^a^	33/232	1.00		62/232	1.00		62/33	1.00	
1–5 years	41/282	1.06	0.63–1.78	97/282	1.01	0.67–1.50	97/41	0.97	0.52–1.80
5–10 years	66/277	1.90	1.16–3.11	141/277	1.55	1.05–2.29	141/66	0.78	0.43–1.41
10+ years	40/132	2.06	1.19–3.57	91/132	1.89	1.22–2.93	91/40	1.07	0.56–2.02
*Trend*			*P*=*0.001*			*P*<*0.0001*			*P*=*0.8*
									
*Time since last use of OC*									
Never^a^	33/232	1.00		62/232	1.00		62/33	1.00	
⩽5 years	92/383	2.14	1.31–3.50	221/383	1.62	1.11–2.35	221/92	0.86	0.48–1.52
5+ years	55/308	1.18	0.72–1.93	108/308	1.12	0.76–1.65	108/55	0.99	0.55–1.79
*Heterogeneity between groups within ever users*			*P*=*0.009*			*P*=*0.02*			*P*=*0.8*
									
*Duration by time since last use of OC*									
*⩽5 years since last use*									
Never^a^	33/232	1.00		62/232	1.00		62/33	1.00	
1–5 years	15/94	1.53	0.71–3.34	29/94	0.73	0.41–1.31	29/15	0.52	0.21–1.28
5+ years	77/289	2.18	1.28–3.73	192/289	1.66	1.11–2.48	192/77	0.88	0.47–1.66
*Trend*			*P*=*0.004*			*P*=*0.001*			*P*=*0.8*
									
*>5 years since last use*									
Never^a^	33/232	1.00		62/232	1.00		62/33	1.00	
1–5 years	26/188	1.09	0.60–1.98	68/188	1.19	0.76–1.86	68/26	1.15	0.56–2.38
5+ years	29/120	1.75	0.96–3.18	40/120	1.22	0.73–2.03	40/29	0.65	0.31–1.37
*Trend*			*P*=*0.06*			*P*=*0.5*			*P*=*0.2*
									
*Duration of combined OC use*^b^									
Never^a^	34/237	1.00		66/237	1.00		66/34	1.00	
1–5 years	42/293	1.03	0.62–1.72	102/293	0.98	0.66–1.44	102/42	0.92	0.49–1.69
5–10 years	66/279	1.81	1.11–2.95	137/279	1.35	0.92–1.98	137/66	0.71	0.40–1.29
10+ years	36/110	2.19	1.25–3.85	81/110	1.84	1.18–2.89	81/36	1.02	0.53–1.96
*Trend*			*P*=*0.001*			*P*=*0.001*			*P*=*0.991*
									
*Ever use of progestagen-only OCs*^b^,^c^									
Never^a^	163/864	1.00		359/864	1.00		359/163	1.00	
Ever	15/55	1.28	0.68–2.43	27/55	1.27	0.74–2.18	27/15	0.75	0.36–1.58
									
*Use of barrier method with regular partners*^d^,^e^									
Never	73/303	1.00		176/303	1.00		176/73	1.00	
Ever	107/616	0.87	(0.61–1.24)	215/616	0.77	(0.58–1.02)	215/107	0.79	(0.52–1.25)

OR_a_, odds ratio for adenocarcinoma *vs* controls; OR_b_, odds ratio for squamous cell carcinoma *vs* controls; OR_c_, odds ratio for squamous cell carcinoma cases *vs* adenocarcinoma cases; all adjusted for age, region, total number of sexual partners, age at first intercourse, smoking, number of negative screening results and education.

a ‘Never’ use includes use for less than I year.

b Excluding those who only used an unknown OC.

c Additionally adjusted for duration of combined OC use.

d Limited to women with at least one regular partner.

e Additionally adjusted for duration of OC use.

). This association was seen for both squamous cell and adenocarcinoma. For squamous cell carcinoma, ever use of oral contraceptives was associated with a marginally significant increase in risk (OR=1.37 (0.97–1.94)), with a similar increase seen in past (OR=1.31 (0.92 –1.87)) and current users (OR=1.64 (1.06–2.55)); for adenocarcinoma, risk was significantly increased in ever users (OR=1.56 (1.01–2.42) and in current, but not in past, users (OR for current users=2.36 (1.34–4.16)). For both types of cancer, there was a clear relationship between cancer risk and duration of use of oral contraceptives, with highly significant trends of increasing risk with increasing duration of use (*P* trend=0.001 for adenocarcinoma and <0.0001 for squamous cell carcinoma). Compared with never use (including use for less than a year), odds ratios for 10 or more years of use were 1.89 (1.22–2.93) for squamous cell carcinoma and 2.06 (1.19–3.57) for adenocarcinoma. Risk was also related to time since last use of oral contraceptives, with higher risk for more recent use. When duration of use was considered in relation to time since last use, a significant increase in cancer risk with increased duration of use of oral contraceptives was seen only in recent users. Over 97% of oral contraceptive users among controls had used combined oral contraceptives, and analysis restricted to use of combined oral contraceptives showed an effect for duration of use very similar to that for all oral contraceptives. There was no association between ever use of progestagen-only oral contraceptives and risk of squamous cell or adenocarcinoma in analyses adjusted for the duration of use of combined oral contraceptives; these analyses are based on limited numbers. Direct comparison showed no significant differences between squamous cell and adenocarcinoma in relation to oral contraceptive use.

### Barrier contraceptives

Information on use of barrier methods of contraception was available only for women with regular sexual partners. Ever use of barrier contraceptives was associated with a decreased risk of cervical cancer, which for squamous cell carcinoma was of marginal statistical significance (ORs for ever use compared with never use=0.77 (0.58–1.02) for squamous cell carcinoma and 0.87 (0.61–1.24) for adenocarcinoma). Twelve women in the study reported using barrier methods always with all regular sexual partners; all of these women were controls.

### Smoking

The prevalence of ever smoking was 57% among control women. Ever smoking was not associated with the risk of squamous cell carcinoma (OR=1.05 (0.79–1.40) or of adenocarcinoma (OR=0.80 (0.56–1.13)). For squamous cell carcinoma, the results suggest that the risk of cancer was higher for current smokers (OR=1.26 (0.93–1.71)) than for ex-smokers (OR=0.70 (0.47–1.03)). The risk of squamous cell carcinoma was significantly increased in long-term (20 or more years) smokers, with an odds ratio of 2.05 (1.29–3.26) and a trend for duration of smoking of borderline significance (*P* trend=0.05). For adenocarcinoma, no association was seen between cancer risk and duration of smoking. No association was found between either type of cervical cancer and intensity of smoking or the age at which smoking started. Direct comparison between squamous cell carcinoma and adenocarcinoma cases showed a consistently higher risk associated with smoking for squamous cell carcinoma. The difference between cancer types was statistically significant for current smoking (OR for squamous cell compared with adenocarcinoma=1.74 (1.11–2.71) and for duration of smoking (OR=2.44 (1.26–4.72) for smoking duration of 20+ years, with a statistically significant trend in relation to duration of smoking (*P* trend=0.04)). This difference is due both to increased risk for squamous cell carcinoma and to decreased risk (with odds ratios of around 0.8) for adenocarcinoma (Table 4

**Table 4:** Odds ratios (ORs) and 95% confidence intervals (CIs) for adenocarcinomas and squamous cell carcinomas of the cervix in relation to smoking

	**Adenocarcinoma**			**Squamous cell carcinoma**			**Squamous *vs* adenocarcinoma**		
	**Cases/controls**	**OR_a_**	**95% CI**	**Cases/controls**	**OR_b_**	**95% CI**	**Squamous/adeno cases**	**OR_c_**	**95% CI**
*Smoking status*									
Never	72/394	1.00		121/394	1.00		121/72	1.00	
Ever	108/529	0.80	0.56–1.13	270/529	1.05	0.79–1.40	270/108	1.50	0.99–2.27
Past	32/181	0.75	0.46–1.20	60/181	0.70	0.47–1.03	60/32	1.05	0.60–1.85
Current	76/348	0.82	0.56–1.21	210/348	1.26	0.93–1.71	210/76	1.74	1.11–2.71
									
*Age started smoking*									
Never	72/394	1.00		121/394	1.00		121/72	1.00	
10–16 years	60/300	0.71	0.47–1.08	165/300	1.07	0.77–1.47	165/60	1.68	1.04–2.72
17+ years	48/229	0.91	0.59–1.38	105/229	1.04	0.74–1.47	105/48	1.31	0.80–2.16
*Heterogeneity between groups within ever smokers*			*P*=*0.1*			*P*=*0.8*			*P*=*0.06*
									
*Total duration for current smokers*									
Never/<1 month	72/394	1.00		121/394	1.00		121/72	1.00	
⩽10 years	8/43	0.85	0.34–2.12	22/43	0.69	0.35–1.37	22/8	1.08	0.40–2.91
10–20 years	37/192	0.72	0.44–1.18	116/192	1.13	0.79–1.62	116/37	1.66	0.96–2.85
20+ years	31/113	0.80	0.46–1.39	72/113	2.05	1.29–3.26	72/31	2.44	1.26–4.72
*Trend within current smokers*			*P*=*0.3*			*P*=*0.05*			*P*=*0.04*
									
*Intensity (cigarettes per day) for current smokers*									
Never	72/394	1.00		121/394	1.00		121/72	1.00	
<15	39/194	0.72	0.45–1.15	109/194	1.28	0.90–1.82	109/39	1.92	1.13–3.25
15+	37/154	0.82	0.51–1.34	101/154	1.26	0.86–1.83	101/37	1.68	0.97–2.92
*Heterogeneity between groups within current* *smokers*			*P*=*0.8*			*P*=*0.8*			*P*=*0.9*

OR_a_, odds ratio for adenocarcinoma *vs* controls; OR_b_, odds ratio for squamous cell carcinoma *vs* controls; OR_c_, odds ratio for squamous cell carcinoma cases *vs* adenocarcinoma cases; all adjusted for age, region, total number of sexual partners, age at first intercourse, duration of oral contraceptive use, number of negative screening results and education.

).

## DISCUSSION

This study was designed to provide a direct comparison of risk factors for invasive adenocarcinoma (including adenosquamous carcinoma) and squamous cell carcinoma of the cervix in premenopausal women. For squamous cell carcinoma, increased risk was associated with increased number of regular sexual partners, early age at first intercourse, use of oral contraceptives, high parity, early age at first birth and long-term smoking. Adenocarcinoma risk was associated with the number of regular sexual partners, early age at first intercourse, oral contraceptive use and early age at first birth. No significant associations were seen between parity or smoking and the risk of adenocarcinoma. The difference between squamous cell and adenocarcinoma in relation to smoking was statistically significant.

Adenocarcinomas and adenosquamous carcinomas of the cervix account for about 15% of invasive cervical cancers, and both absolute and relative numbers of adenocarcinomas in screened populations have increased in recent years. This may reflect a cohort effect similar to that seen for squamous cell carcinomas and related to increased exposure to HPV infection in women born since 1960 (Madeleine *et al*, 2001; Sasieni and Adams, 2001), and the fact that cervical screening may be less effective in detecting adenocarcinomas than squamous cell carcinomas (Clarke and Anderson, 1979; Mitchell *et al*, 1995; Bergstrom *et al*, 1999). Their relative rarity has limited attempts to define risk factors for adenocarcinomas and adenosquamous carcinomas of the cervix (Silcocks *et al*, 1987; Parazzini and La Vecchia, 1990; Kjaer and Brinton, 1993), but several recent controlled studies with relatively large numbers (over 100) of adenocarcinoma cases have provided clearer evidence (Ursin *et al*, 1994,^1996^; Bjorge and Kravdal, 1996; Thomas and Ray, 1996; Lacey *et al*, 1999,^2000^,^2001^; Madeleine *et al*, 2001; Munoz *et al*, 2002; Altekruse *et al*, 2003). Of these studies, four (one cohort study (Bjorge and Kravdal, 1996) and three multicentre case–control studies from WHO (WHO, 1993; Thomas and Ray, 1995), IARC (Munoz *et al*, 2002) and the USA (Lacey *et al*, 1999,^2000^,^2001^ ; Altekruse *et al*, 2003)) have directly compared risk factors for adenocarcinoma and squamous cell carcinoma. While many risk factors appear to be common to both types of cervical cancer, it has been suggested that adenocarcinomas of the cervix may also show similarities with adenocarcinomas of the endometrium (Kjaer and Brinton, 1993; Altekruse *et al*, 2003).

While there is strong evidence that HPV infection is the major factor in the development of adenocarcinomas and adenosquamous carcinomas as well as of squamous cell carcinomas of the cervix, adenocarcinomas have been associated particularly with HPV type 18 and related virus types, unlike squamous cell carcinomas in which in most populations HPV 16 and related types predominate (Clifford *et al*, 2003). A number of studies have found a lower prevalence of HPV infection associated with adenocarcinomas (70–80%) than with squamous cell carcinomas (virtually all of which are associated with HPV using recent PCR methods) (Andersson *et al*, 2001; Lo *et al*, 2002; Clifford *et al*, 2003). It is not clear whether this represents a real difference between histological types, or possible misclassification of some adenomatous tumours. Other established risk factors for cervical cancer may affect exposure to HPV infection (e.g. number of sexual partners) or may influence the outcome of HPV infection (e.g. oral contraceptive use (Moreno *et al*, 2002; Green *et al*, 2003; Smith *et al*, 2003), reproductive factors such as parity (Munoz *et al*, 2002) and possibly smoking (Plummer *et al*, in press)).

Sexual behaviour, and in particular the number of sexual partners, is strongly associated both with HPV infection and with the risk of squamous cell carcinoma of the cervix. Most controlled studies have also found the risk of cervical adenocarcinoma to increase directly with the lifetime number of sexual partners (Brinton *et al*, 1987; Parazzini *et al*, 1988; Ursin *et al*, 1996; Chichareon *et al*, 1998; Madeleine *et al*, 2001; Altekruse *et al*, 2003), and studies providing a direct comparison have found no differences in risk between adenocarcinoma and squamous cell carcinoma in all and in HPV-positive women (Brinton *et al*, 1987; Horowitz *et al*, 1988; Brinton *et al*, 1993; Chichareon *et al*, 1998; Ngelangel *et al*, 1998). Our results are consistent with existing evidence. In our study the risk of both types of cervical carcinoma was related only to the number of regular sexual partners; similar results have also been reported in two previous studies (Brinton *et al*, 1987; Herrero *et al*, 1990) and may reflect the need for repeated exposure to the HPV virus for persistent infection to be established. Early age at first intercourse, while clearly related to lifetime number of partners, is generally considered to be an independent risk factor for squamous cell carcinoma of the cervix (Herrero *et al*, 1990; Cuzick *et al*, 1996; Deacon *et al*, 2000), and we found age at first intercourse to be a relatively strong independent risk factor for both adenocarcinoma and squamous cell carcinoma in the present study. Previous studies of adenocarcinoma of the cervix have generally found no association between age at first intercourse and cancer risk in analyses adjusted for the number of sexual partners (Brinton *et al*, 1987; Brinton *et al*, 1993; Ursin *et al*, 1996; Chichareon *et al*, 1998; Ngelangel *et al*, 1998; Altekruse *et al*, 2003).

In this study, both high parity and early age at first birth were associated with the risk of squamous cell carcinoma of the cervix; adenocarcinoma risk was associated with early age at first birth but not with parity. Munoz *et al* (2002), in a pooled analysis of 10 case–control studies in HPV-positive women, found that the risk of squamous cell carcinoma increased with the number of full-term pregnancies; there was no equivalent trend in the risk of adenocarcinoma and adenosquamous carcinoma, although the risk of adenocarcinoma was higher in parous women than in nulliparous. Most other case–control studies, including those taking HPV infection into account, have shown an association with parity for squamous cell carcinoma of the cervix (Brinton *et al*, 1987,^1993^; Parazzini *et al*, 1988; Hildesheim *et al*, 2001; Altekruse *et al*, 2003) but, with the exception of one study (Parazzini *et al*, 1988), not for adenocarcinoma (Brinton *et al*, 1987,^1993^; Ursin *et al*, 1996). Altekruse *et al* (2003) found an inverse relationship between parity and cervical adenocarcinoma risk. No association with parity for either adenocarcinoma or squamous cell carcinoma of the cervix was found in two cohort studies (Kvale *et al*, 1988; Bjorge and Kravdal, 1996). Case–case studies have generally found adenocarcinoma to be more strongly associated than squamous cell carcinoma with nulliparity (Tasker and Collins 1974; Korhonen, 1980; Milsom and Friberg 1983; Silcocks *et al*, 1987), but these studies are difficult to interpret as most of them lack adequate adjustment for potential confounding factors. Previous studies of cervical cancer have found age at first birth to be an independent risk factor for squamous cell (Munoz *et al*, 2002), but not for adenocarcinoma (Kvale *et al*, 1988; Parazzini *et al*, 1988, Bjorge and Kravdal, 1996; Ursin *et al*, 1996; Munoz *et al*, 2002; Altekruse *et al*, 2003).

The use of barrier methods of contraception has been associated with decreased risk both of preinvasive cervical lesions and of invasive (largely squamous) cervical cancers (Manhart and Koutsky, 2002), and this association has been found in studies restricted to HPV-positive women (Kjaer *et al*, 1996, Coker *et al*, 2001, Hildesheim *et al*, 2001). The evidence, although suggestive of a protective effect of barrier contraception, is not entirely consistent and interpretation is severely limited in most studies by the lack of detailed reliable evidence on contraceptive use. There is limited evidence for a decreased risk of adenocarcinoma associated with the use of barrier methods (Ursin *et al*, 1996; Altekruse *et al*, 2003). Our results, adjusted for sexual behaviour and for oral contraceptive use, are consistent with a reduced risk of both types of cervical cancer in barrier contraceptive users. It is interesting that all of the 12 women in this study who reported using barrier contraceptives always with all (regular) partners were among the controls.

Oral contraceptive use is a well-established risk factor for cervical cancer; recent analyses of case–control and cohort studies have confirmed that risk for both squamous cell and adenocarcinoma is directly related to the duration of use of oral contraceptives in all and in HPV-positive women, (Moreno *et al*, 2002; Smith *et al*, 2003). The effect of oral contraceptives on cancer risk is strongest for current and recent use and decreases with time since last use. Our results are entirely consistent with these previous studies and show clearly the inter-relationship between duration of use and time since last use of oral contraceptives for both adenocarcinoma and squamous cell carcinoma. Previously published results from the UK National Case–Control Study of Cervical Cancer (based on a subset of the cases and controls included in this analysis) have shown that the association between risk of cervical cancer and duration of use of oral contraceptives in this study is also seen in analyses restricted to HPV-positive women (Berrington *et al*, 2002). As in other studies, the majority of oral contraceptive users in the present study had used combined oral contraceptives and our results for ever use and for duration of use were similar for all oral contraceptives and for combined oral contraceptives only. We found no significant association between the ever use of progestagen-only oral contraceptives and risk of adenocarcinoma or squamous cell carcinoma of the cervix in analyses adjusted for duration of oral contraceptive use. These results are, however, based on small numbers and the ORs are not incompatible with those for ever use of all (very largely, combined) oral contraceptives. Use of progestagen-only injectable contraceptives may be associated with a small increase in the risk of cervical cancer (Smith *et al*, 2003); it is not clear whether this is related to histology (Thomas and Ray, 1995).

We found evidence for an association between duration of smoking and the risk of squamous cell carcinoma, but no significant association between smoking and adenocarcinoma risk. This is consistent with the results of previous studies (Plummer *et al*, in press). No study has reported a significant association between smoking and the risk of adenocarcinoma of the cervix (Brinton *et al*, 1987; Parazzini *et al*, 1988; Ursin *et al*, 1996; Chichareon *et al*, 1998; Ngelangel *et al*, 1998; Lacey *et al*, 2001; Madeleine *et al*, 2001) and of four case–control studies directly comparing risk for adenocarcinoma and squamous cell carcinoma, smoking was found to be a significant risk factor for squamous cell carcinoma but not for adenocarcinoma in two (Brinton *et al*, 1987, Ngelangel *et al*, 1998), with no statistically significant association between smoking and cervical cancer in the other two studies (Chichareon *et al*, 1998; Lacey *et al*, 2001). In the study by Lacey *et al*, the results suggested that smoking may increase the risk of squamous cell carcinoma, but decrease the risk of adenocarcinoma (with odds ratios for ever smoking of 1.6 (0.9–2.9) for squamous cell carcinoma and 0.6 (0.3–1.1) for adenocarcinoma). In our analysis, smoking was also consistently associated with an increased risk of squamous cell carcinoma and a decreased risk of adenocarcinoma. Further evidence is needed to clarify the relationship between smoking and different histological types of cervical cancer, but it appears that smoking may be a risk factor for squamous cell and not for adenocarcinoma. A greater effect of smoking on the risk of squamous cell carcinoma would be consistent with the pattern of risk identified for some other types of epithelial cancer, for example, cancers of the oesophagus, of the nasal cavity and possibly of the lung (IARC, in press).

Other postulated risk factors for carcinomas of the cervix include socioeconomic status and body weight. While we found no association between educational level and either type of cancer in the present study, there is consistent evidence from controlled studies that the risk both of adenocarcinoma and of squamous cell carcinoma of the cervix is higher in women of low socioeconomic status (Parikh *et al*, 2003). Early reports from case–case studies suggesting that squamous cell and adenocarcinomas differed in relation to socioeconomic status may have been due to confounding by factors such as smoking. Four out of five case–control studies with information on body weight have reported an increased risk of adenocarcinoma of the cervix in women with high body mass index (Parazzini *et al*, 1988; Lacey *et al*, 2001), weight (Brinton *et al*, 1987; Lacey *et al*, 2001) or weight gain in adult life (Ursin 96), with no association for squamous cell carcinoma where a direct comparison was made (Brinton *et al*, 1987; Lacey *et al*, 2001). The remaining study found no evidence of an association between weight and risk of adenocarcinoma or squamous cell carcinoma (Brinton *et al*, 1993), and no consistent difference was found in body weight between women with adenocarcinoma and those with squamous cell carcinoma in case–case studies (Korhonen, 1980; Milsom and Friberg, 1983; Honore *et al*, 1991; Hopkins and Morley, 1991). There was no evidence in the present study of an association between squamous cell or adenocarcinoma and self-reported body weight. Evidence relating to body weight is difficult to interpret because of limitations in the measures of weight used, and, in some studies, in the degree of adjustment for confounding factors.

The suggestion that adenocarcinomas of the uterine cervix may be more closely related in aetiology to adenocarcinomas of the endometrium than to squamous cell carcinomas of the cervix was based initially on reports from case–case and small case–control studies. These suggested that cervical adenocarcinomas shared many of the risk factors associated with endometrial carcinoma, including obesity, nulliparity, diabetes, hypertension and a lack of (or even inverse) relationship with smoking. More recent evidence discussed here does not on the whole support this view. There is clear evidence that strong risk factors such as HPV infection, sexual activity and oral contraceptive use are common to both histological types of cervical cancer (and show no or, in the case of oral contraceptive use, an inverse relationship with endometrial cancer); and no good evidence that squamous cell and adenocarcinomas of the cervix differ in relation to body weight, socioeconomic status, diabetes or hypertension. Unlike endometrial cancer, neither type of cervical cancer is related to age at menarche or age at menopause. The relationship between parity and adenocarcinoma of the cervix is less clear than that seen for squamous cell carcinoma, but the suggestion that adenocarcinomas resemble endometrial carcinomas (which show an inverse relationship with parity) in this respect has not been confirmed. The only consistent difference between cervical squamous cell and adenocarcinomas appears to be in relation to smoking.

Our study has a number of limitations, many of which apply also to other published studies. We were unable to take HPV status into account directly in the present analyses, although adjustment for number of sexual partners should to some extent act as a surrogate for adjustment for HPV. We combined cases of adenocarcinoma and of adenosquamous carcinoma in order to obtain larger numbers of these relatively rare cancers. However, risk factors for adenocarcinoma and adenosquamous carcinoma may differ (Brinton *et al*, 1987) and ideally they should be considered separately. There is also the possibility that some tumours may have been misclassified. We used histological diagnoses from medical records; in comparable studies, up to 5% of recorded adenocarcinomas were reclassified on histological review (Lacey *et al*, 1999; Madeleine *et al*, 2001). It has been suggested that ‘contamination’ of cervical adenocarcinomas with adenocarcinomas arising in the endometrium could account for differences observed between cervical squamous cell and adenocarcinomas. However, in this study of premenopausal women, misclassification of endometrial adenocarcinomas is very unlikely as endometrial cancer is predominantly a disease of postmenopausal women. It should also be borne in mind that a substantial proportion of women diagnosed with cervical adenocarcinoma may have concurrent squamous cell carcinomas (Brinton *et al*, 1993, Madeleine *et al*, 2001).

The main strengths of our study are the relatively large numbers of women with adenocarcinoma, giving the study sufficient power to detect equivalent risks for both types of carcinoma (the study had 80% power at 95% significance to detect individual risk factor ORs of between 1.5 and 2.0 for risk factor prevalences between 10 and 80%, for both squamous cell and adenocarcinoma), and the fact that we were able to adjust for major potential confounding factors in the analyses. This study provides information on risk factors for cervical cancer in young women, a relatively little studied group in whom the incidence of adenocarcinoma of the cervix has increased in recent years. Our findings are generally consistent with those from a previous study of squamous cell carcinoma of the cervix in young women in the UK (Cuzick *et al*, 1996), in particular, in the importance of sexual behaviour as a risk factor and in the lack of effect of socioeconomic status on risk.

This study provides evidence to support the view that squamous cell and adenocarcinomas of the cervix share many risk factors, including those likely to be related to exposure to HPV infection, such as sexual behaviour, and those, such as oral contraceptive use, more likely to affect the outcome of HPV infection. Smoking, however, while clearly related positively to the risk of squamous cell carcinoma was not (or even, possibly, inversely) related to the risk of adenocarcinoma. This finding is consistent with those from other studies. Further information is needed to confirm these results.
